# Radial pseudoaneurysm following coronary angiography in a patient with autosomal dominant polycystic kidney disease

**DOI:** 10.1007/s00392-023-02159-7

**Published:** 2023-01-23

**Authors:** Insa E. Emrich, Michael Böhm, Felix Mahfoud

**Affiliations:** Department of Internal Medicine III, Cardiology, Angiology, Intensive Care Medicine, Saarland University Medical Center, Kirrberger Straße 1, IMED, 66421 Homburg, Germany

Sirs,

An 80-year-old woman presented to the emergency room with an asymptomatic, pulsatile swelling of her right wrist 4 weeks following coronary angiography via the transradial route. The procedure was performed using a 6-French sheath, which was removed and followed by manual and 4 h of TR-band^®^ compression. During the procedure, the standard fixed intra-arterial dose of 5000 IU of unfractionated heparin (UFH) was administered without adjustment for weight or monitoring. Neither activated clotting time (ACT) nor partial thromboplastin time (PTT) was measured before or after the procedure. At the time of admission, the swelling had the size of a chicken egg (~ 4.5 × 2.5 cm). According to the patient, the swelling steadily increased in size through the last 4 weeks. At the time point of admission, the patient received antiplatelet therapy consisting of aspirin 100 mg and clopidogrel 75 mg once daily, respectively. There was no indication for oral anticoagulation therapy.

The patient’s motor function, vascularization, and sensitivity of the right hand were intact. A consecutively performed ultrasonography validated the clinical diagnosis of a radial pseudoaneurysm (Fig. 1). Due to its enormous size, the woman underwent surgical excision and suture of the right radial artery immediately. The postoperative course was uneventful and follow-up visits revealed robust radial artery pulses with no residual abnormalities.

**Fig. 1:**
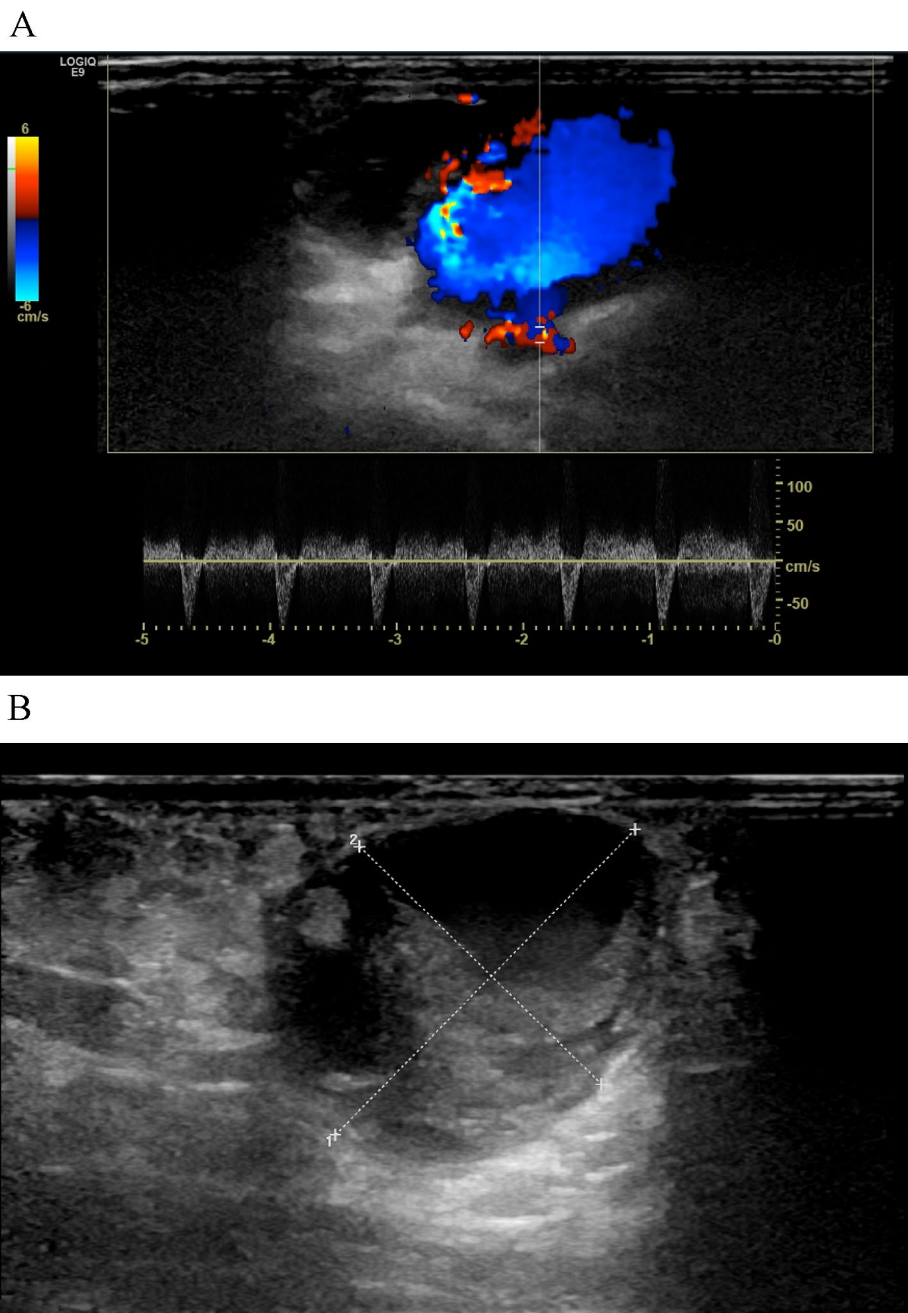
**A** Depiction of the pseudoaneurysm (longitudinal view) outgoing from the right radial artery with characteristic to-and-fro waveform by pulse Doppler. **B** Doppler ultrasound of the pseudoaneurysm. Aneurysmal sac dimension of 29.5 × 24.0 × 26.0 mm with marginal thrombosis

The patient had a history of end-stage kidney disease, secondary to autosomal dominant polycystic kidney disease (ADPKD). After several years of peritoneal dialysis, she successfully underwent kidney transplant in her right pelvis in 2015. Due to progressively decreasing graft function, 6 weeks before her presentation to our department, she underwent angiography and stent implantation of the right common iliac artery to improve perfusion of the renal graft’s artery (anastomosis of renal artery to the right common iliac artery; eGFRcr-cys: 42 ml/min/1.73 m^2^ at the time point of intervention). During the procedure, 5000 IU UFH was administered without periprocedural ACT or PTT monitoring. Following sheath removal, AngioSeal^®^ 6F placement, manual compression for several minutes and femoral pressure bandages over 6 h, a severe haemorrhage due to a leak of the right common femoral artery occurred, which required immediate surgical vascular repair. During post-surgical monitoring, the patient suffered from a non-ST elevation myocardial infarction, and hence a coronary angiography was performed.

Pseudoaneurysms, also called false aneurysm, of the radial artery are rare [1]. In general, important risk factors associated with the occurrence of pseudoaneurysms are the use of large-bore sheaths, periprocedural anticoagulation, antiplatelet therapy, vascular infection, or multiple punctures at a single site [2].

Our patient developed two severe vascular complications after arterial intervention in close succession. Although the patient had various comorbidities, ADPKD appears to be the most relevant risk factor.

ADPKD represents the most common hereditary kidney disease [3]. Mutations in genes *PKD1* (*located on chromosome 16p13.3*) and *PKD2 (located on chromosome 4q22.1)* can be found in most cases [4]. Besides complications due to cysts’ expansion (e.g. haematuria, infections or nephrolithiasis), affected patients often present with extra-renal manifestation, as vascular abnormalities, particularly intracranial aneurysms. In approximately 10% of asymptomatic ADPKD patients, intracranial aneurysms can be found [5]. In addition, case reports and smaller studies describe dissection and aneurysm in almost every other (larger) artery. Clinical data revealed that patients with ADPKD do not only have an increased risk of developing intracranial aneurysm, but also face a higher risk of bleeding, embolic infarction, and carotid artery dissection during intracranial aneurysm treatment [6]. Unfortunately, systematic research of vascular abnormalities in ADPKD patients, apart from intracranial aneurysm, are sparse. One possible explanation for the high prevalence of vascular complications is linked to mutations in *PKD1* and *PKD2* [7]. Analysis of homozygous mutations of *PKD1* and *PKD2* in mice showed increased subcutaneous oedema, focal vascular leaks, and haemorrhage [8, 9]. In addition, in mice with hypomorphic *PKD1* allele, aneurysm formation of the descending thoracic and abdominal aorta was observed [10]. Further rodent data suggest haploinsufficiency for *PKD1* and *PKD2,* followed by altered intracellular calcium homeostasis*,* leading to a variety of cellular and vascular defects [11, 12]. Ameku et al. noticed that the expression level of the matrix metalloproteinase (MMP) 1 was specifically elevated in induced pluripotent stem cells-derived endothelia from ADPKD patients with intracranial aneurysm compared to those without intracranial aneurysm [13]. This observation was confirmed in 354 ADPDK patients showing a close correlation between serum MMP1 levels and the development of intracranial aneurysm [13]. However, our understanding of the underlying pathogenesis remains limited [5].

In summary, we present a rare case of a pseudoaneurysm of the radial artery after coronary angiography in a patient with ADPKD. Since patients with ADPKD have a higher risk of vascular complications, close attention should be paid to detect potential access site complications following interventional procedures.

## Data Availability

The data are available on request from the corresponding author (IEE).
